# The Human Vulvar Microbiome: A Systematic Review

**DOI:** 10.3390/microorganisms9122568

**Published:** 2021-12-12

**Authors:** Lisa Pagan, Roos A. M. Ederveen, Bertine W. Huisman, Jan W. Schoones, Romy D. Zwittink, Frank H. J. Schuren, Robert Rissmann, Jurgen M. J. Piek, Mariëtte I. E. van Poelgeest

**Affiliations:** 1 Centre for Human Drug Research, 2333 CL Leiden, The Netherlands; lpagan@chdr.nl (L.P.); bhuisman@chdr.nl (B.W.H.); m.i.e.van_poelgeest@lumc.nl (M.I.E.v.P.); 2 Department of Gynecology and Obstetrics, Leiden University Medical Center, Albinusdreef 2, 2333 ZA Leiden, The Netherlands; 3 Faculty of Health, Medicine and Life Sciences, Maastricht University Medical Centre, 6229 ER Maastricht, The Netherlands; roosederveen@hotmail.com; 4 Department of Obstetrics and Gynaecology and Catharina Cancer Institute, Catharina Ziekenhuis, Michelangelolaan 2, 5623 EJ Eindhoven, The Netherlands; jurgen.piek@catarinaziekenhuis.nl; 5 Directorate of Research Policy (Formerly: Walaeus Library), Leiden University Medical Center, Albinusdreef 2, 2333 ZA Leiden, The Netherlands; j.w.schoones@lumc.nl; 6 Center for Microbiome Analyses and Therapeutics, Leiden University Medical Center, Albinusdreef 2, 2333 ZA Leiden, The Netherlands; r.d.zwittink@lumc.nl; 7 Netherlands Organisation for Applied Scientific Research, TNO, 3704 HE Zeist, The Netherlands; frank.schuren@tno.nl; 8 Leiden Skin Institute, 2333 CL Leiden, The Netherlands; 9 Leiden Amsterdam Center for Drug Research, Leiden University, 2300 RA Leiden, The Netherlands

**Keywords:** microbial targets, cancer development, vulvar microbiome, vaginal microbiome, drug development

## Abstract

The link between cancer and the microbiome is a fast-moving field in research. There is little knowledge on the microbiome in ((pre)malignant) conditions of the vulvar skin. This systematic review aims to provide an overview of the literature regarding the microbiome composition of the healthy vulvar skin and in (pre)malignant vulvar disease. This study was performed according to the PRISMA guidelines. A comprehensive, electronic search strategy was used to identify original research articles (updated September 2021). The inclusion criteria were articles using culture-independent methods for microbiome profiling of the vulvar region. Ten articles were included. The bacterial composition of the vulva consists of several genera including *Lactobacillus*, *Corynebacterium*, *Staphylococcus* and *Prevotella*, suggesting that the vulvar microbiome composition shows similarities with the corresponding vaginal milieu. However, the vulvar microbiome generally displayed higher diversity with commensals of cutaneous and fecal origin. This is the first systematic review that investigates the relationship between microbiome and vulvar (pre)malignant disease. There are limited data and the level of evidence is low with limitations in study size, population diversity and methodology. Nevertheless, the vulvar microbiome represents a promising field for exploring potential links for disease etiology and targets for therapy.

## 1. Introduction

The human skin is a complex barrier organ and consists of a symbiosis between host tissue and a large aggregate of microorganisms including bacteria, viruses, and fungi, known as microbiota. The microbiome is the composition of all microbial taxa and their genes within a community [1]. The human microbiome plays a key role in health and has been linked to several disease conditions, including inflammatory diseases, skin conditions and cancer [2,3,4]. Following recent publications in solid cancer types and their precursors, the link between the development of cancer and the microbiome is a fast-moving field in the area of cancer research [5,6,7]. Several micro-organisms are well-known for their oncogenic potential, such as human papillomavirus (HPV) in cervical carcinoma and *Helicobacter pylori* in gastric cancer, prompting effective and targeted vaccine and treatment rollout [8,9]. Recent studies suggest that the microbiome could influence carcinogenesis through dysregulation of inflammation, immunity and metabolism [10]. Furthermore, the microbiome may influence cancer therapy delivery and response [11,12]. A useful avenue would be to investigate the associations between cancer and the microbiome in a range of (pre)malignant diseases. Knowledge of the healthy microbiome composition is of paramount importance before any oncogenic associations can be identified.

The incidence rate of vulvar squamous cell carcinoma (VSCC) is 1 to 2 per 100,000 and increases with age [13]. Currently, a HPV-dependent and an HPV-independent pathway in developing VSCC have been identified [14,15,16]. HPV-related premalignant vulvar lesions are commonly caused by HPV16 or HPV18 and referred to as high-grade squamous intraepithelial lesions (HSIL) [15]. Chronic inflammatory conditions, such as lichen sclerosus (LS) and lichen planus, may predispose VSCC and its precursor, differentiated vulvar intraepithelial neoplasia (dVIN). However, the mechanisms for malignant progression remain largely unknown [16,17]. There is a considerable amount of taboo associated with vulvar disease, adding to underreporting, lack of clinical recognition and treatment [18]. Considerable physical, sexual and psychological morbidity is caused by vulvar (pre)malignant diseases [19,20,21].

Elucidation of the healthy cervicovaginal microbiota composition and its changes that may correlate to gynecological and obstetric disease is ongoing. Several studies have investigated the role of vaginal microbiome changes to the development of cervical dysplasia and cervical cancer [22,23,24]. A number of studies have documented the vulvar microbiome composition [25]. However, these are of limited scope. No comprehensive overview of the current knowledge of the vulvar microbiome is available. Although the causal pathway between HPV and HSIL is well understood, no other associations between the microbiome and emergence of vulvar disease have been described. The objective of this systematic review of the literature is to identify and summarize studies investigating the composition and changes of the vulvar microbiome in health and disease. In addition, suggestions for the design of future research will be provided. Improving the knowledge on the vulvar microbiome may contribute to new perspectives in the management of (pre)malignant vulvar disease.

## 2. Materials and Methods

This systematic review adheres to the relevant criteria from the PRISMA statement (Preferred Reporting Items for Systematic Reviews and Meta-Analyses) [26]. The methods used, including identification, screening, eligibility and inclusion, were agreed by the authors and the protocol was registered with PROSPERO (reference number CRD42020181148) [27,28]. The scope of this review was altered to focus on the vulvar microbiome composition rather than the complete gynecological field.

### 2.1. Search Strategy

Relevant scientific papers were identified by a systematic online cross-database search performed in April 2020 (last update 29 September 2021), using PubMed, Embase, Emcare, Web of Science, Cochrane Library and Academic Search Premier. Search strategies for all databases were adapted from the PubMed strategy and developed with assistance of an experienced librarian of the Walaeus Library of the Leiden University Medical Center (JS). The search strategy consisted of the medical subject headings and text words related to the following anatomical sites: vulva, vagina, anus and cervix in (a) the following disease entities: high-grade squamous intraepithelial lesions (HSIL), low-grade squamous intraepithelial lesions (LSIL), carcinoma, lichen sclerosus, lichen planus and differentiated VIN (dVIN) and (b) healthy individuals. See Supplementary File S1 for the complete search strategies for each database.

The search was restricted to English language and human studies. In addition, hand searching of the reference lists of relevant reviews and included studies was undertaken to identify additional relevant references.

### 2.2. Inclusion Criteria

Original articles examining the vulvar microbiome through culture-independent methods in tissue or swabs in patients with vulvar/cervical HSIL, LSIL (including genital warts), vulvar and anal squamous cell carcinoma, lichen sclerosus, lichen planus, dVIN and healthy women were included. As the majority of the human microbiome is uncultivable and potentially misleading results may arise from cultivation studies, the study was restricted to research articles in which culture-independent techniques using molecular methods with DNA were applied for microbiota profiling DNA [29,30,31,32].

### 2.3. Exclusion Criteria

Culture-based studies, animal studies, (systematic) reviews, conference abstracts, articles written in languages other than English and case reports were excluded. Additionally, studies that did not include a culture-independent microbiome analysis of the vulvar region were excluded.

### 2.4. Study Selection

Titles and abstracts of studies retrieved were screened independently using the Rayyan online tool [33] by two review authors (LP and RE) to identify studies that met the inclusion criteria outlined above. The full text of these potentially eligible studies was retrieved and independently assessed for eligibility by two review authors (LP and RE). Disagreement over the eligibility of specific studies between the two review authors was resolved through discussion with a third review author (MvP).

### 2.5. Data Extraction

Data were extracted from the included studies for assessment of study quality and evidence synthesis. Predefined extracted information included: study setting (including country); study population; participant demographics and baseline characteristics; target organ; target disease; in- and exclusion criteria; study methodology (including sample collection method and type of microbiome analysis) and microbiome outcomes (including community types and main findings). Synthesis of the summarizing figure was based on the reported relative abundance of the bacterial composition, when applicable.

### 2.6. Assessment of Risk of Bias and Level of Evidence

Two review authors (LS and RE) independently assessed the risk of bias and the level of evidence according to the Critical Appraisal Checklist characteristics as recommended by the Joanna Briggs Institute [34]. The level of evidence was assessed as recommended by Grading of Recommendations, Assessment, Development and Evaluation (GRADE) guidelines [35]. Disagreements in the risk of bias and level of evidence assessment were resolved by discussion between the review authors with involvement of a third review author (MvP).

## 3. Results

### 3.1. Number of Retrieved Papers

**Figure 1:**
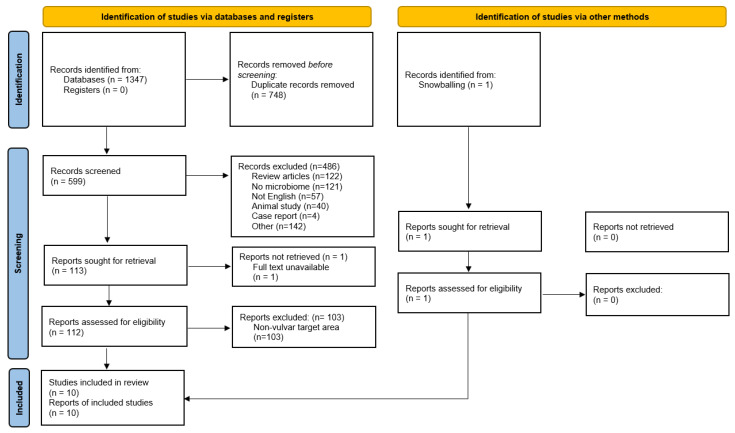
Flow chart of the study.

A total of 1347 articles were obtained from the initial database search. After removal of 748 duplicates, 599 records were screened for title and abstract, from which 486 were excluded (Figure 1). From this search, 113 articles were assessed for eligibility based on the full text, of which nine were selected to be included in the qualitative synthesis of this review. In addition, one study was identified through snowballing and included in the review. The excluded full text articles were mostly excluded because these focused on a different area (i.e., vagina without vulvar data) or used a different, inappropriate technique for microbiome assessment (culture-dependent techniques or cytokine assays). One recent study by Park et al. combined sampling of the vestibulum and vagina and was therefore excluded from the synthesis of results [36].

From the ten studies that were included in this review, nine studies employed 16S rRNA gene amplicon sequencing for analysis of the vulvar microbiome. The remaining study, by Miyamoto et al., employed qPCR analysis using five genus or species-specific bacterial primers [37]. One study, by Bruning et al., also analyzed the fungal fraction of the microbiota through ITS amplicon sequencing [38]. In total, vulvar microbiome analysis has been performed on 261 women (total data set). No studies used shotgun metagenomics or other culture-independent techniques that could identify the microbial composition of the vulva. All included studies are summarized in Table 1.

### 3.2. The Healthy Vulvar Microbiome

Seven out of ten studies investigated the vulvar microbiota in healthy women or factors that can influence the healthy microbiota composition.

Brown et al. aimed to characterize the vulvar microbiome of four healthy Caucasian women aged 28–44 years by 16S rRNA gene amplicon sequencing analysis from single vaginal, labia majora and labia minora samples [39]. In three out of four women, the most abundant phylotypes on the labia minora and labia majora were *Lactobacillus crispatus* or *L. iners*, whilst the fourth participant’s labia minora and majora were dominated almost equally by *L. iners* (resp. 32.0 and 28.3%), *Atopobium vaginae* (resp. 26.8 and 17.5%) and *Megasphaera elsdenii* (resp. 30.1 and 12.5%), species that are known colonizers of the vagina. The authors concluded that the dominant phylotypes from the vulva were also dominant members of communities in the corresponding vaginal samples, which were published in a separate paper [40]. Furthermore, communities found on the labia majora were generally more diverse than those found on the labia minora, with two to fourteen times more phylotypes detected on the former location. *Staphylococcus epidermidis* and *Corynebacterium* are phylotypes of cutaneous origin that were found on the labia majora but not, excluding one case, on the labia minora. Emerging from the intestinal tract, *Enterococcus faecalis* was found in a higher proportion on the labia majora than on the labia minora.

Bruning et al. performed 16S rRNA gene and ITS amplicon sequencing on 34 and 16 labia majora samples, respectively, in a clinical trial to a microbiome-friendly vulvar wash in a Caucasian population aged 18–55 years. In follow-up samples throughout the study, it was found that the wash had no effect on the bacterial and fungal microbiota composition. The predominant bacterial genera found at baseline (relative abundance) included *Corynebacterium* (27–47%)*, Lactobacillus* (12–18%), *Staphylococcus* (4–10%)*, Prevotella* (4–12%), *Propionibacterium* (1–13%) and *Finegoldia* (3–5%). Several genera belonging to *Actinobacteria* were present in low relative abundance (<1%). The predominant fungi were *Cryptococcus* (20–50%), *Malassezia* (0.3–21%)*, Saccharomyces* (20%)*, Cladosporium* (1–12%) and *Rhodotorula* (2%). The vulvar pH was found to be significantly influenced by relative abundance of *Lactobacillus*. The authors hypothesize that the colonization of the identified organisms could correlate to the heterogeneous vulvar skin structure and function, such as *Staphylococcus* and *Corynebacterium* on moist areas, *Propionibacterium* and *Malassezia* on sebaceous skin and other *Actinobacteria spp*. on dry areas.

The only study included in this review to employ species-specific primers for qPCR, by Miyamoto et al., found a significantly (*p* < 0.001) higher total bacterial load on the labia and groin compared to the mons pubis or the inner thigh in a cross-sectional sample of 40 healthy Japanese women aged 20–40 years [37]. *Lactobacillus spp.* and *Staphylococcus epidermidis* were identified as the dominant species at all sites. *Lactobacillus spp.* were found in a statistically (*p* < 0.001) higher abundance on the labia and groin compared to the mons pubis and the inner thigh. *Staphylococcus aureus* was found in 60% of women, while the abundance was significantly (*p* < 0.001) higher on the labia and groin compared to the other two regions. *Prevotella spp.*, a collection of species commonly found in the gastrointestinal tract, were only detected on the labia and groin samples, although prevalent in 95% of participants. *Propionibacterium acnes* was identified on the labia of all subjects, and in 98% of samples of the mons pubis and inner thigh.

Costello et al. performed a longitudinal study in nine healthy adults in the United States of America (USA, ethnicity undisclosed), including three women aged 30–35, on the bacterial community of up to 27 body sites with samples of the labia minora on two consecutive days [41]. The authors concluded that observed variation between samples was mostly explained by the different body sites sampled, followed by differences between individuals and by changes over time. The microbial community on the labia minora clustered separately from the rest of the samples identified in this study, mostly because of dominance of *Lactobacillus* (48.6%), probably arising from the vagina. Additionally, presence of *Prevotella* (16%) and *Finegoldia* (8.9%) on the labia minora were described. The remaining various other species (26.4%) were present in low abundance or not present in all subjects in the study.

### 3.3. The Association of the Menstrual Cycle and Obesity and the Vulvar Microbiome

One study, by Shiraishi et al., evaluated the effect of menstruation on the vulvar microbiota in ten healthy Japanese women aged 31–43 years [42]. No bacterial species were found to be consistently increased or decreased in abundance before or during menstruation when sampled once one week before menstruation and once on the second day of menstruation. Seven out of ten women presented with a microbiota of the labia minora that predominantly consisted of by *L. crispatus* or *L. iners* and remained so during menstruation. In five of these women, this remained so during menstruation, while the dominant species switched during menstruation in two subjects, *L. iners* to *L. crispatus* and vice versa. The vaginal microbiome was also determined in three out of ten participants. They concluded that the vaginal samples displayed highly similar species as found on the vulva.

Hickey et al. performed a prospective study to investigate the effect of menarche on the composition of the vulvar and vaginal microbiome in 32 healthy 10–13 year-old girls with different ethnicities [43]. Quarter-yearly swabs were collected up to three years. During the study, 67.7% (21 out of 32) of the participants reached menarche. They concluded that the vulvar and vaginal microbiota composition of pre- and perimenarchal girls had a high *Lactobacillus* abundance. There was a moderately high degree of concordance between the vulva and the vagina, although the vulva tended to have a greater variety of bacterial taxa. Specifically, *Segniliparus, Murdochiella* and *Fusobacterium* showed a stronger association with the vulva in relative abundance levels compared to the vagina.

One cross-sectional study by Vongsa et al. focused on the differences in vulvar microbiota composition in 20 obese women (body mass index > 30) compared to 20 lean women (body mass index 18–25) aged 18–35 years (ethnicity undisclosed) [44]. Kruskal–Wallis tests were used to calculate differences in relative abundance of bacterial genera. The authors concluded that women with a high BMI have a distinct vulvar microbial pattern compared to average-weight women (*p* = 0.005). *Lactobacillus* spp. were more prevalent on the vulva of lean women than of obese women (*p* = 0.00), whereas *Corynebacterium* spp. (*p* = 0.04) and *Anaerococcus* spp. (*p* = 0.01) were more prevalent on the vulva of obese women. They also found that the community populations of the labia majora clustered distinctly from the labia minora in obese women (*p* = 0.001). The diversity did not differ between the labia minora and the labia majora. *Finegoldia* and *Lactobacillus* were more prevalent on the labia minora (resp. *p* = 0.02 and *p* = 0.05). Conversely, *Corynebacterium* was more prevalent on the labia majora compared to the labia minora (*p* = 0.00), as was *Staphylococcus* (*p* = 0.00).

### 3.4. The Microbiome in Vulvovaginal Disease

Two studies investigated the role of the microbiome in vulvar vestibulitis or provoked vestibulodynia, comparing patients to controls [45,46]. The first, by Jayaram et al., found no significant differences in the vulvar or vaginal bacterial microbiota composition between 15 cases (mean age 30.8) and 20 healthy women (mean age 32.6) [46]. Additionally, corresponding vestibular and vaginal samples were grossly similar in composition, with no significant differences in prevalence and dominance of species. The dominant genera in vestibular samples of patients with vestibulodynia were *Lactobacillus* (76.7%), *Streptococcus* (10%), *Gardnerella* (6.7%), *Anaerococcus* (3.3%) and *Enterococcus* (3.3%). The dominant genera in vestibular samples of healthy controls were *Lactobacillus* (80%) and *Gardnerella* (20%). On species level, *L. crispatus* and *L. iners* were most often dominant in the vulvar and vaginal samples of both patients and controls. *L. coleohominis* was prevalent on the vestibular samples of patients (10%) and controls (13.3%), but not identified in their vaginal samples. *L. gasseri* was identified in vestibular (36.7%) and vaginal (26.7%) samples of patients with vestibulodynia, but not in healthy controls.

Murina et al. observed no significant differences in bacterial composition in vaginal and vestibular samples in 20 women with provoked vestibulodynia (PVD) compared to 18 healthy controls, both groups comprising of Caucasian women aged 23–48 years [45]. *L. gasseri* was identified as the dominant vulvar species in the PVD group, but not as the dominant species in healthy women, whilst correlating with pain and dyspareunia intensity (*p* < 0.001). The most prevalent genera in the women with PVD were *Lactobacillus* (80.9%), *Gardnerella* (9.5.%) and *Atopobium* (9.5%). In the control group, the most dominant genera were *Lactobacillus* (64.7%)*, Gardnerella* (11.7%) and *Bifidobacterium* (5.8%). The vestibular samples displayed no statistically different bacterial composition compared to vaginal samples.

Finally, one recent study by Chattopadhyay et al. tried to elucidate the vulvar and gut microbiome of five premenarchal girls with lichen sclerosus (LS) and five girls with non-specific vulvovaginitis as compared to three healthy girls in a case-control study [47]. The mean age of the population was 6 years and the population was of mixed ethnicity. They found that in vulvar samples, 26 bacterial genera or species were significantly different (*p* < 0.05) between LS, non-specific vulvovaginitis and healthy controls. Specifically, girls with LS and non-specific vulvovaginitis presented with a lower relative abundance of *Streptococcus angionosus*, but a higher abundance of *Peptostreptococcus anaerobius* and *Prevotella melaninogenica* compared to controls. In fecal samples, 21 bacterial genera or species were identified as significantly different (*p* < 0.005) between LS, non-specific vulvovaginitis and healthy controls. Girls with LS showed a higher abundance of *Dialister* spp., *Clostridiales*, *Paraprevotella* spp. and *E. coli* compared to healthy controls, while *Phascolarctobacterium* spp. was present in a lower abundance. This study identified an overlap of 34 genera or species present in both the fecal and vulvar milieu, suggesting exchange between the microbial niches.

## 4. Discussion

To our knowledge, this is the first study that comprehensively assessed the vulvar microbiome in health and (pre)malignant vulvar disease. One of the main findings of this study is that there is very limited knowledge on the vulvar microbiome. The bacterial genera and species that have been described on the healthy vulva are several taxa of *Lactobacillus, Corynebacterium*, *Staphylococcus* and *Prevotella*, suggesting possible emergence from vaginal, cutaneous and intestinal origin. The results of this review suggest that the vulva may constitute a separate microbial niche with different signatures found on various anatomical sites within the vulva, e.g., labia minora, labia majora and mons pubis. However, only ten studies in a total of 261 women have been conducted in heterogenous study designs and populations, therefore this picture is far from complete.

*Lactobacillus* are well-known lactic-acid-producing colonizers of the female genital tract, maintaining an acidic vaginal milieu. It has been suggested that *Lactobacillus* dominance plays a protective role against cervical dysplasia, although the true nature of this association is not fully elucidated [48,49]. In this review, dominance of *L. crispatus* and *L. iners* was observed on the vulva in most healthy women. *L. gasseri* dominance was noted only in a proportion of patients with vestibulodynia, but not in the healthy control groups, although this correlation was not considered statistically significant in either studies [45,46]. *Corynebacterium* species are commonly found cutaneous bacteria [50]. *Corynebacterium* presence has been described on the vulva in serval studies, although in most cases not as the dominant species or in specific correlation to vulvar disease. *Corynebacterium* has been described as an occasional colonizer of the vaginal tract, specifically when Lactobacillus abundance is low [51]. *Staphylococcus aureus*, from the phylum *Firmicutes*, is found predominantly on the skin and in the upper respiratory tract. In this review, *S. aureus* prevalence on the vulva ranged from 0 [39] to 63% [37]. From literature, *S. aureus* colonization of the vagina is 9.2% [52], with similar varying rates (6.8 to 67%) have been observed on the external female genitalia [25,53]. *Prevotella*, often found in the gut, has been associated with periodontal disease [54]. Vaginal *Prevotella* presence has been associated with bacterial vaginosis, but also with a healthy vaginal environment [55]. Notably on the vulva, *Prevotella* was reported in 95% of labia and groin samples by Miyamoto et al., whilst only 1 in 10 labia minora samples from Shiraishi et al. showed presence of *Prevotella* [37,42].

**Figure 2:**
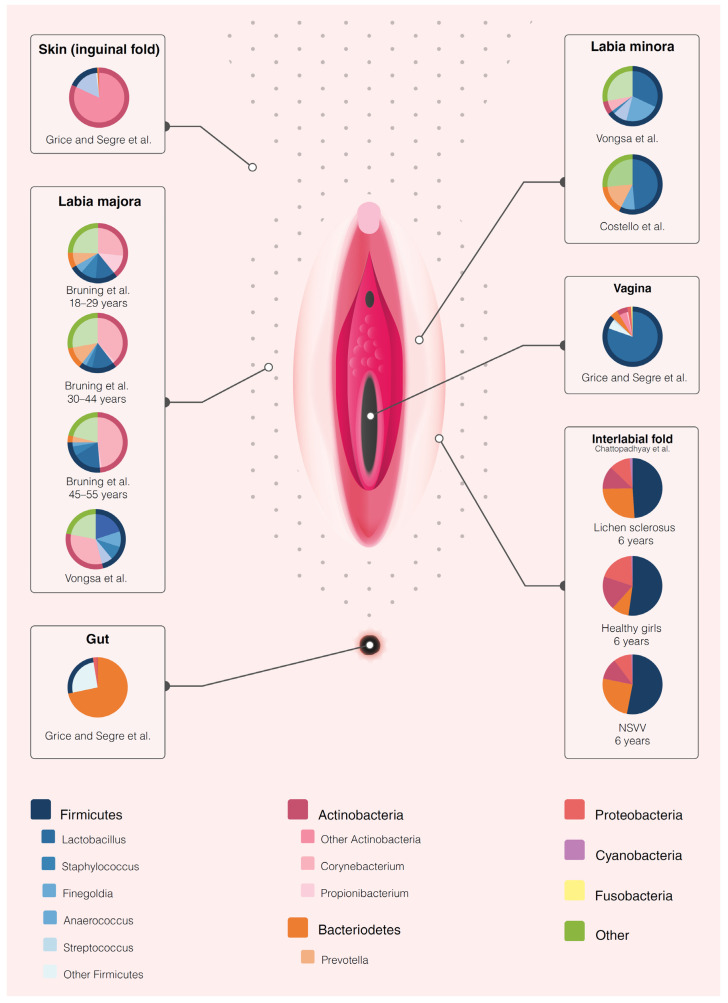
Graphical representation of the vulvar microbiome composition. The microbiome composition of the vulva appears to potentially be distinct from the microbiome composition of the adjacent anatomical sites (vagina, gut or skin). It should be noted that this figure was based on only four studies that reported the relative abundance on phylum or genus level. The remaining studies did not have a report on the relative abundance nor raw data available in the public domain that could allow for generation of relative abundance data. The outer circle represents the reported phyla per study per anatomical location upon the vulva. If applicable, the inner circle represents the genera reported in the same study. The data on the microbiome composition of the vagina, gut and skin (inguinal fold) was adapted from Grice and Segre, 2011 and 2012 [50,56].

Visualization of the vulvar microbiota is provided in Figure 2, comparing the vulvar microbiome composition with those of vaginal, intestinal and cutaneous (inguinal fold) body sites [50,56]. Only four out of ten studies could be incorporated in the depiction due to heterogeneous reporting and lack of raw data availability. Although data are scarce and study populations are heterogenous, we hypothesize that the vulva could constitute an inward-facing gradual transition zone from predominantly cutaneous commensals towards components of the vaginal microbiome with intestinal influences. Micro-organisms thrive on body sites that supply the optimal growth conditions, including pH, nutrients, oxygenation and moisturization [57]. The heterogeneous vulvar skin composes an occluded, humid environment with friction and areas with and without keratinization, challenging the interpretation of the observations [58,59]. Higher bacterial loads are found on occluded areas of the skin (inguinal fold, axilla, postauricular), which was also observed on the vulva by Miyamoto et al. [37]. High relative abundance of *Corynebacteria* has been described in moist and sebaceous areas such as the inguinal fold [50], which may correlate with the observation of their distinct presence on the labia majora. There are several potentially confounding factors in this representation of the vulvar microbiota. Firstly, sequencing techniques measure DNA, meaning that no distinction can be made between live and dead bacteria. Additionally, these techniques sensitively pick up overflow or contamination from vaginal or intestinal sites, which are more densely populated than skin sites. This can swiftly cause an overrepresentation of these contaminating bacteria on vulvar sites. Finally, the data for the current visualization of the vulvar microbiome constitution has been deducted from small, heterogeneous study populations sampled in varying study designs, thus the picture is in its infancy and these current associations may prove to be incidental and need larger confirmatory studies to become generalizable.

One of the focus points of this review was to identify existing correlations of the vulvar microbiome and (pre)malignant vulvar disease. Only one study reported on premenarchal LS in girls. Chattopadhyay et al. correlate the identified higher abundance of *Dialister* spp. and lower abundance of *Roseburia faecis* in the gut of LS patients to findings in other inflammatory diseases such as ankylosing spondyloarthritis and Crohn’s disease. The authors also argue that their LS patients display a more dysbiotic vulvar microbiome, with a possible excess of *Prevotella* spp., *Porphyromonas* spp. and *Parvimonas* spp., correlating it to observations of these taxa in chronic periodontitis. The observed higher abundance of *Peptostreptococcus* spp., *Prevotella* spp. in girls with LS has previously been described in psoriatic lesions [60]. Likewise, higher abundance of *Porphyromonas* spp. and *Parvimonas* spp. have also been found in hidradenitis suppurativa [61], which was also observed in girls with LS. However, this pilot study was only performed in five LS cases and three controls in a pediatric population. This leaves many questions about the interplay between the vulvar microbiome and LS, including adult women with LS or other vulvar (pre)malignancies.

Previous research in microbiome perturbations in cervicovaginal or male genital (pre)malignant disease may generate hypotheses for vulvar disease. A recent meta-analysis by Norenhag et al. found that an increasing stage of HPV-driven cervical dysplasia was associated with a higher prevalence of a non-*Lactobacillus* dominated vaginal microbiome [22]. In addition, several studies have reported an increased prevalence of *Snaethia* spp. in the vaginal microbiome of patients with hrHPV infections, cervical intraepithelial neoplasia and invasive cervical carcinoma [23,62,63,64]. Likewise, *Mycoplasma* spp. is reportedly often found to co-infect with hrHPV [65]. Of note, many of these reports were cross-sectional studies describing results of one or two stages of cervical dysplasia, with few longitudinal trials that allow for validation of this relationship. In Nigerian men with anal cancer, Nowak et al. found that *Sneathia* spp. was associated with HPV-16 prevalence among men who have sex with men with HIV or at risk for HIV [66]. In penile cancer, Onywera et al. have described a higher a greater relative abundance of *Prevotella*, *Peptinophilus* and *Dialister* and lower relative abundance of *Corynebacterium* in hrHPV-infected men [67]. Cohen et al. found that the urine bacteriome of male LS patients with showed enrichment of *Bacillales*, *Bacteriodales* and *Pasteurellales* [68]. Increased incidence of Epstein-Barr virus (EBV) in LS biopsy tissue has been described in both female (26.5%) and male (37–38.3%) LS patients [69,70,71]. A recent meta-analysis found a positive correlation between EBV and oral lichen planus (odds ratio 4.41) [72]. EBV is a known cause for Burkitt lymphoma, nasopharyngeal carcinoma and diffuse large B-cell lymphoma and other lymphoma subtypes [73,74,75]. These findings may provide a lead for the currently unknown etiology of LS. However, no studies in this review have investigated the vulvar virome nor the microbiome composition of adult LS patients.

Several limitations of the current literature can be identified. Firstly, the sample size of all studies is low, with a minimum of three and a maximum of 45 participants per study, which does not allow for robust results within a highly variable field of research. Furthermore, all studies of the adult vulvar microbiome were only performed in Caucasian or Japanese participants, while two studies omit disclosure of ethnicity data [41,44]. The only two studies that do include a diverse population (e.g., Black, Hispanic, Native American) were carried out in young girls [43,47]. As the vaginal bacteriota is known to differ across ethnic groups [76,77,78,79,80] and this review suggests parallels between vaginal and vulvar samples, it is imperative to include more diverse populations in future studies. Another limitation is the lack of elaboration and elucidation of other potentially confounding factors. For instance, the current literature only includes women aged 6–55 years. The only study to LS was conducted in young girls and there is no literature on the microbiome composition in the adult group at risk for vulvar (pre)malignant conditions. In some of the included publications, e.g., by Murina et al., Vongsa et al. and Chattopadhyay et al. [44,45,47], highly significant findings were found in extremely small study populations without corrections for multiple testing or described considerations to avoid bias. Results from these studies should therefore be carefully interpreted and the potential risk for type I errors ought to be noted for future research.

Lifestyle choices should also be considered, such as vulvovaginal hygiene, vaginal douching or drying practices or type and frequency of sexual intercourse. Patient characteristics that may alter the microbiome are insufficiently considered in the current literature. These aspects include, but are not limited to, ethnicity, age, weight, hormonal state and systemic (immunosuppressive) disease. Antibiotic use is the only factor listed as an exclusion criterium in all but one study, with other restrictions applied haphazardly when comparing the included studies (Supplementary Table S1). Furthermore, there is a lack of longitudinal studies, with only four studies that sample the vulvar microbiome at more than one time point [38,41,42,43]. Research has shown that the composition of the healthy vaginal microbiome can easily be disrupted but appears to be stable over a longer period of time [51,81]. Sampling at a single time point is only a snapshot representation and disregards dynamics of the microbial ecosystem in the pathway of disease onset and progression. Co-occurrence of certain microbial dysbiosis and disease states at a single time point cannot unveil the direction of association.

There is little information on the presence and function of viruses, parasites and fungi on the vulva. Nine out of ten studies analyzed the bacteriome through 16S rRNA gene amplicon sequencing. The choice of a certain 16S rRNA region (i.e., hypervariable region V1–V3 or V3–V4) can lead to heterogeneity in quantification of certain species and influence classification level. It is currently recognized that V1–V3 of the 16s rRNA gene correlates most with shotgun metagenomics for cutaneous and vaginal analysis and is preferred over targeting of V3–V4 [82,83]. Two studies included in this review used primers for the V3–V4 region [42,47], and in two studies the targeted region could not be traced [39,44]. Only one study included fungal analysis [38] and no studies included shotgun metagenomics sequencing. Furthermore, there was a large discrepancy in sampling methods (dry or wet swab, scrub) between studies, which can greatly affect the outcomes of microbiota profiling [84]. Every minor variation picked up at sampling is subsequently amplified by molecular microbiome assay techniques. In low biomass samples, such as those of the vulva, negative control samples should also be added to the analysis. Only one out of ten studies in this review reported the use of negative or blank control samples [47]. The limitations of the analysis techniques for the identification of the microbiome and sampling methods employed in the current studies contribute to the low level of evidence and difficulties in comparison of the presented results.

We recommend longitudinal, case-control study designs for future vulvar microbiome research in a range of (pre)malignant vulvar diseases and in healthy controls. Ideally, shotgun metagenomics methods should be chosen over solely 16S rRNA gene amplicon sequencing to allow for a more complete picture of the microbiome and its functional potential. We urge including samples from several anatomical locations within the vulva, in addition to vaginal, intestinal or cutaneous samples to allow for intra-individual comparison of results. As the current knowledge of the vulvar microbiome is centralized around data from premenopausal Caucasian women, it is advised to attempt to recruit a more diverse population in future studies. If possible, lifestyle factors that could disrupt microbiome results (e.g., sexual activities, topical medication or emollient use, washing, hair removal practices) prior to microbiome sampling should be standardized or recorded. Hormonal or menstrual cycle status may also influence results status. Many questions remain on the composition of the healthy vulvar microbiome and the role of the microbiome in the origin and progression of vulvar disease.

## 5. Conclusions

This systematic review investigates the role of the microbiome in vulvar health and disease for the first time. We conclude that there is very limited knowledge on the microbiome of the vulva. There are indications that microbiota composition of the vulva shows many similarities with the corresponding vaginal milieu, although the vulvar microbiome generally showed a higher diversity with commensals of cutaneous and fecal origin, potentially giving the vulva a unique signature that ought to be further elucidated in further studies.

No studies have been performed to the microbiome of (pre)malignant vulvar disease. Future studies unraveling the vulvar microbiome in much greater phylogenetic detail and with frequent longitudinal information are highly needed for better understanding of disease and to identify potential novel biomarkers for diagnosis and disease monitoring.

## Figures and Tables

**Table 1:** Summary table of the studies included in the review HV = healthy volunteers; USA = United States of America; rRNA= ribosomal RNA; qPCR = quantitative polymerase chain reaction; BMI = body mass index; VVS = vulvar vestibulitis.

	Author, Year	Focus	Study Subjects	Subject Age	Ethnicity + Country	Sample Locations	Sample Type	Microbial Analysis	Key Findings	Limitations	Risk of Bias	Level of Evidence
Health and the influence of patient factors	Brown et al, 2007	Healthy	4 HV	28–44 years	Caucasian, USA	Labia minora, labia majora, vagina	Scrape samples	16S rRNA amplification of unspecified region	Bacteriome vulva ≈ vagina. Dominant vulvar species: 3/4 L. *crispatus* or *L. iners*, 1/4 *L. iners*, *Atopobium vaginae* and *Megaspheara elsdenii*.	Small sample size. No longitudinal sampling. No specification of sequencing region.	Low	Very low
	Bruning et al, 2020	Healthy	36 HV	18–55 years	Caucasian, USA	Labia majora	Modified liquid cup scrub method	16S rRNA amplification of the V1–V3 region and fungal ITS sequencing	Bacterial relative abundance at baseline: 27–47% *Corynebacterium*, 12–18% *Lactobacillus*, 4–10% *Staphylococcus*, 3–12% *Prevotella*, 1–13% *Propionibacterium* and 3–5% *Finegoldia*. Fungal relative abundance at baseline: 20–50% *Cryptococcus*, 0.3–21% *Malassezia*, 1–12% *Cladosporium* and 2% *Rhodoturula*	Focus reporting on effects investigational product, not microbiome. 34/36 bacterial, 16/36 fungal samples showed amplification for analysis.	Low	Low
	Miyamoto et al, 2013	Healthy	40 HV	20–40 years	Japanese, Japan	Labia majora, groin, mons pubis, inner thigh	Saline wetted sterile swabs	qPCR for specific genera (*S. epidermidis, S. aureus, P. acnes*, *Lactobacilli* spp., *Prevotella* spp.)	Labia + groin vs. mons pubis/inner thigh: ↑ *Lactobacilli* and *S. aureus*. *Prevotella* spp. on labia + groin only.	No extensive sequencing data due to employed procedure. No longitudinal sampling.	Low	Very low
	Costello et al, 2009	Healthy	3 HV	30–35 years	Unknown, USA	Labia minora	NaCl + Tween wetted sterile swabs	16S rRNA amplification of the V2 region	Predominant taxa: *Lactobacillus* (48.6%)¸ *Prevotella* (16%) and *Finegoldia* (8.9%)	Small sample size. Focus not on vulvar microbiome, but on other body sites.	Low	Very low
	Shiraishi et al, 2010	Menstruation	10 HV	31–43 years	Japanese, Japan	Labia minora, vagina (3/10)	Scrape samples	16S rRNA amplification of the V3–V4 region	No species consistently changed abundance before or during menstruation. 7/10 showed predominance of *L. crispatus* or *L. iners*	Small sample size. Only 3/10 vaginal cross–reference samples. No longitudinal sampling.	Low	Very low
	Hickey et al, 2015	Menarche	32 HV	10–12.9 years	Mixed Black, Caucasian, Native American, Hispanic. USA	Labia minora, vagina	Dry, sterile flocked swabs	16S rRNA amplification of the V1–V3 region	Bacteriome vulva ≈ vagina (mean more similarities before menarche. Greater variety bacterial taxa on vulva compared to the vagina. Abundance lactic acid producing bacteria increases with puberty on vulva and vagina	Focus on vaginal microbiome. No report comparing the pre- and post-menarchal vulvar microbiome	Low	Low
	Vongsa et al, 2019	Obesity	20 obese (BMI >30) 20 HV (BMI 18-25)	18–35 years	Unknown, USA	Labia majora, labia minora	Swab	16S rRNA amplification of unspecified region	Obese vs. HV: ↑ *Corynebacerium* spp. and *Anaerococcus* spp., ↓ *Lactobacillus* spp. Labia majora more diverse than labia minora.	No longitudinal sampling. No ethnicity data disclosure. No specification of sequencing region.	Low	Low
Disease	Jayaram et al, 2014	Vulvar vestibulitis syndrome (VVS)	20 VVS 15 HV	Mean 30.8 (VVS) and 32.6 (HV) years	Caucasian, USA	Vestibulum, vagina	Swab	16S rRNA amplification of the V1–V3 region	No differences vulvar or vaginal bacteriome composition cases vs. controls. Bacteriome vestibulum ≈ vagina. Most prevalent VVS: *Lactobacillus, Gardnerella, Atopobium*. Most prevalent HV: *Lactobacillus, Streptococcus* and *Gardnerella*	No longitudinal sampling.	Low	Low
	Murina et al, 2020	Provoked vestibulodynia (PVD)	20 PVD 18 HV	23–48 years	Caucasian, Italy	Vestibulum, vagina	Swab	16S rRNA amplification of the V3 region	*L. gasseri* only dominant in PVD Most prevalent genera PVD: *Lactobacillus, Gardnerella* and *Atopobium*. Most prevalent genera HV: *Lactobacillus, Gardnerella* and *Bifidobacetrium*.	No longitudinal sampling.	Low	Low
	Chattopadhyay et al, 2021	Pre-menarchal lichen sclerosus	5 LS 5 NSVV 3 HV	Mean 6 years	Mixed Caucasian, Black and Hispanic, USA	Labial fold, perineum, feces	Dry flocked swabs	16S rRNA amplification of the V3–V4 region	LS vs. HV: vulvar bacteriome ↑ *Porphyromonas* spp., *Parvimonas* spp., *Peptoniphilus* spp. *Prevotella* spp., *Dialister* spp. and ↓ *Peptostreptococcus* spp. *Corynebacterium* spp. Faecal bacteriome LS: ↑ *Dialister* spp.	Small sample size No longitudinal sampling Only premenarchal girls No species level determination due to employed procedure.	Low	Very low

## Data Availability

The study protocol is available at PROSPERO (reference number CRD42020181148). The search strategy is available in Supplementary file S1. The extracted data from the included studies can be found in Table 1, Figure 2 and Supplementary Table S1. Other files are available from the authors upon request.

## Supplementary Materials

The following are available online at https://www.mdpi.com/article/10.3390/microorganisms9122568/s1: Supplemental Table S1: Summary of lifestyle rules; Supplementary File S1: Search strategy.
