# Supplementary material for: The Human Vulvar Microbiome: A Systematic Review
Source: Microorganisms. 2021 Dec 12;9(12):2568. doi: 10.3390/microorganisms9122568 (PMC8705571; doi:10.3390/microorganisms9122568)
Supplement: Supplementary file 1 [file microorganisms-09-02568-s001.zip › Supplementary File 1_Search strategy.pdf]

PubMed

((((("Microbiota"[mesh] OR "microbiota"[tw] OR microbiota*[tw] OR "microbiome"[tw] OR microbiom*[tw] OR "Microbial Community"[tw] OR "Microbial Communities"[tw] OR "Microbial Consortia"[tw] OR "Mycobiome"[tw] OR Mycobiom*[tw] OR "Fungal Community"[tw] OR "Fungal Communities"[tw] OR "bacterial flora"[tw] OR "microbial flora"[tw] OR "Bacteria/growth and development"[Mesh]) AND ("Vulva"[mesh] OR "vulva"[tw] OR "vulvas"[tw] OR "vulvar"[tw] OR vulva*[tw] OR "Bartholin's Glands"[tw] OR "Bartholin's Gland"[tw] OR "Clitoris"[tw] OR "Labia minora"[tw] OR "Labia minor"[tw] OR "Labia majora"[tw] OR "Labia major"[tw] OR "Vagina"[mesh] OR "vagina"[tw] OR "vaginas"[tw] OR "vaginal"[tw])) OR "vaginal microbial communities"[tw] OR "vaginal microbial community"[tw] OR "vaginal microbial composition"[tw] OR "vaginal microbial diversity"[tw] OR "vaginal microbial ecology"[tw] OR "vaginal microbial ecosystem"[tw] OR "vaginal microbial flora"[tw] OR "vaginal microbiological ecosystem"[tw] OR "vaginal microbiological flora"[tw] OR "vaginal microbiology"[tw] OR "vaginal microbiome"[tw] OR "vaginal microbiomes"[tw] OR "vaginal microbiota"[tw] OR "vaginal microbiotas"[tw]) AND ("Lichen Sclerosus et Atrophicus"[Mesh] OR "Vulvar Lichen Sclerosus"[Mesh] OR "Lichen sclerosus"[tw] OR "Lichen planus"[mesh] OR "Lichen planus"[tw] OR "Penile Neoplasms"[Mesh] OR "Anus Neoplasms"[Mesh] OR "Vulvar Neoplasms"[Mesh] OR "Vaginal Neoplasms"[Mesh] OR "Uterine Cervical Neoplasms"[Mesh] OR (("Penile"[tw] OR "penis"[tw] OR "anal"[tw] OR "anus"[tw] OR "vulva"[tw] OR "vulvar"[tw] OR "vagina"[tw] OR "vaginal"[tw] OR "cervix"[tw] OR "cervical"[tw]) AND ("cancer"[tw] OR "cancers"[tw] OR "carcinoma"[tw] OR "carcinomas"[tw] OR "adenocarcinoma"[tw] OR "adenocarcinomas"[tw] OR "tumor"[tw] OR "tumour"[tw] OR "tumors"[tw] OR "tumours"[tw] OR "neoplasm"[tw] OR "neoplasms"[tw] OR "malignancy"[tw] OR "malignancies"[tw])) OR "Papillomavirus Infections"[Mesh] OR Papillomavir*[tw] OR "HPV"[tw] OR HPV*[tw] OR "Squamous Intraepithelial Lesions"[Mesh] OR "HSIL"[tw] OR "uvin"[tw] OR "VaIN"[tw] OR intraepithel*[tw] OR intra-epithel*[tw] OR "CIN"[tw] OR "AIN"[tw] OR "Condylomata Acuminata"[Mesh] OR "Condylomata Acuminata"[tw] OR "genital wart"[tw] OR "genital warts"[tw] OR "Venereal Warts"[tw] OR "Venereal Wart"[tw] OR "low-grade SIL"[tw] OR "LSIL"[tw])) OR (("Microbiota"[mesh] OR "microbiota"[tw] OR microbiota*[tw] OR "microbiome"[tw] OR microbiom*[tw] OR "Microbial Community"[tw] OR "Microbial Communities"[tw] OR "Microbial Consortia"[tw] OR "Mycobiome"[tw] OR Mycobiom*[tw] OR "Fungal Community"[tw] OR "Fungal Communities"[tw] OR "bacterial flora"[tw] OR "microbial flora"[tw] OR "Bacteria/growth and development"[Mesh]) AND ("Vulva"[mesh] OR "vulva"[tw] OR "vulvas"[tw] OR "vulvar"[tw] OR vulva*[tw] OR "Bartholin's Glands"[tw] OR "Bartholin's Gland"[tw] OR "Clitoris"[tw] OR "Labia minora"[tw] OR "Labia minor"[tw] OR "Labia majora"[tw] OR "Labia major"[tw])))

Embase

(((((exp *"Microbiome"/ OR "microbiota".ti,ab OR microbiota*.ti,ab OR "microbiome".ti,ab OR microbiom*.ti,ab OR "Microbial Community".ti,ab OR "Microbial Communities".ti,ab OR "Microbial Consortia".ti,ab OR "Mycobiome".ti,ab OR Mycobiom*.ti,ab OR "Fungal Community".ti,ab OR "Fungal Communities".ti,ab OR *"bacterial flora"/ OR "bacterial flora".ti,ab OR "microbial flora".ti,ab) AND (exp *"Vulva"/ OR "vulva".ti,ab OR "vulvas".ti,ab OR "vulvar".ti,ab OR vulva*.ti,ab OR "Bartholin's Glands".ti,ab OR "Bartholin's Gland".ti,ab OR "Clitoris".ti,ab OR "Labia minora".ti,ab OR "Labia minor".ti,ab OR "Labia majora".ti,ab OR "Labia major".ti,ab OR exp "Vagina"/ OR "vagina".ti,ab OR "vaginas".ti,ab OR "vaginal".ti,ab)) OR "vaginal microbial communities".ti,ab OR "vaginal microbial community".ti,ab OR "vaginal microbial composition".ti,ab OR "vaginal microbial diversity".ti,ab OR "vaginal microbial ecology".ti,ab OR "vaginal microbial ecosystem".ti,ab OR "vaginal microbial flora".ti,ab OR "vaginal microbiological ecosystem".ti,ab OR "vaginal microbiological flora".ti,ab OR "vaginal microbiology".ti,ab OR "vaginal microbiome".ti,ab OR "vaginal microbiomes".ti,ab OR "vaginal microbiota".ti,ab OR "vaginal microbiotas".ti,ab) AND (*"Lichen Sclerosus et Atrophicus"/ OR "Lichen sclerosus".ti,ab OR *"Lichen planus"/ OR "Lichen planus".ti,ab OR exp *"Penile Tumor"/ OR exp *"Anus Tumor"/ OR exp *"Vulva Tumor"/ OR exp *"Vagina Tumor"/ OR exp *"Uterus Tumor"/ OR "cancer".ti,ab OR "cancers".ti,ab OR "carcinoma".ti,ab OR "carcinomas".ti,ab OR "adenocarcinoma".ti,ab OR "adenocarcinomas".ti,ab OR "tumor".ti,ab OR "tumour".ti,ab OR "tumors".ti,ab OR "tumours".ti,ab OR "neoplasm".ti,ab OR "neoplasms".ti,ab OR "malignancy".ti,ab OR "malignancies".ti,ab OR exp *"Papillomavirus Infection"/ OR Papillomavir*.ti,ab OR "HPV".ti,ab OR HPV*.ti,ab OR "HSIL".ti,ab OR "dvin".ti,ab OR "uvin".ti,ab OR "VaIN".ti,ab OR intraepithel*.ti,ab OR intra-epithel*.ti,ab OR "CIN".ti,ab OR "AIN".ti,ab OR exp *"Condyloma Acuminatum"/ OR "Condylomata Acuminata".ti,ab OR "genital wart".ti,ab OR "genital warts".ti,ab OR "Venereal Warts".ti,ab OR "Venereal Wart".ti,ab OR "low-grade SIL".ti,ab OR "LSIL".ti,ab)) OR ((exp *"Microbiome"/ OR "microbiota".ti,ab OR microbiota*.ti,ab OR "microbiome".ti,ab OR microbiom*.ti,ab OR "Microbial Community".ti,ab OR "Microbial Communities".ti,ab OR "Microbial Consortia".ti,ab OR "Mycobiome".ti,ab OR Mycobiom*.ti,ab OR "Fungal Community".ti,ab OR "Fungal Communities".ti,ab OR *"bacterial flora"/ OR "bacterial flora".ti,ab OR "microbial flora".ti,ab) AND (exp *"Vulva"/ OR "vulva".ti,ab OR "vulvas".ti,ab OR "vulvar".ti,ab OR vulva*.ti,ab OR "Bartholin's Glands".ti,ab OR "Bartholin's Gland".ti,ab OR "Clitoris".ti,ab OR "Labia minora".ti,ab OR "Labia minor".ti,ab OR "Labia majora".ti,ab OR "Labia major".ti,ab)))

Web of Science

((((ts=("Microbiome" OR "microbiota" OR microbiota* OR "microbiome" OR microbiom* OR "Microbial Community" OR "Microbial Communities" OR "Microbial Consortia" OR "Mycobiome" OR Mycobiom* OR "Fungal Community" OR "Fungal Communities" OR "bacterial flora" OR "microbial flora") AND ts=("Vulva" OR "vulva" OR "vulvas" OR "vulvar" OR vulva* OR "Bartholin's Glands" OR "Bartholin's Gland" OR "Clitoris" OR "Labia minora" OR "Labia minor" OR "Labia majora" OR "Labia major" OR "Vagina" OR "vagina" OR "vaginas" OR "vaginal")) OR ts=("vaginal

microbial communities" OR "vaginal microbial community" OR "vaginal microbial composition" OR "vaginal microbial diversity" OR "vaginal microbial ecology" OR "vaginal microbial ecosystem" OR "vaginal microbial flora" OR "vaginal microbiological ecosystem" OR "vaginal microbiological flora" OR "vaginal microbiology" OR "vaginal microbiome" OR "vaginal microbiomes" OR "vaginal microbiota" OR "vaginal microbiotas")) AND ts=("Lichen Sclerosus et Atrophicus" OR "Lichen sclerosus" OR "Lichen planus" OR "Lichen planus" OR "Penile Tumor" OR "Anus Tumor" OR exp"Vulva Tumor" OR "Vagina Tumor" OR "Uterus Tumor" OR "cancer" OR "cancers" OR "carcinoma" OR "carcinomas" OR "adenocarcinoma" OR "adenocarcinomas" OR "tumor" OR "tumour" OR "tumors" OR "tumours" OR "neoplasm" OR "neoplasms" OR "malignancy" OR "malignancies" OR "Papillomavirus Infection" OR Papillomavir* OR "HPV" OR HPV* OR "HSIL" OR "uvin" OR "dvin" OR "VaIN" OR intraepithel* OR "intra-epithel*" OR "CIN" OR "AIN" OR "Condyloma Acuminatum" OR "Condylomata Acuminata" OR "genital wart" OR "genital warts" OR "Venereal Warts" OR "Venereal Wart" OR "low-grade SIL" OR "LSIL")) OR (ts=("Microbiome" OR "microbiota" OR microbiota* OR "microbiome" OR microbiom* OR "Microbial Community" OR "Microbial Communities" OR "Microbial Consortia" OR "Mycobiome" OR Mycobiom* OR "Fungal Community" OR "Fungal Communities" OR "bacterial flora" OR "microbial flora") AND ts=("Vulva" OR "vulva" OR "vulvas" OR "vulvar" OR vulva* OR "Bartholin's Glands" OR "Bartholin's Gland" OR "Clitoris" OR "Labia minora" OR "Labia minor" OR "Labia majora" OR "Labia major")))

Cochrane

((((("Microbiome" OR "microbiota" OR microbiota* OR "microbiome" OR microbiom* OR "Microbial Community" OR "Microbial Communities" OR "Microbial Consortia" OR "Mycobiome" OR Mycobiom* OR "Fungal Community" OR "Fungal Communities" OR "bacterial flora" OR "microbial flora") AND ("Vulva" OR "vulva" OR "vulvas" OR "vulvar" OR vulva* OR "Bartholin's Glands" OR "Bartholin's Gland" OR "Clitoris" OR "Labia minora" OR "Labia minor" OR "Labia majora" OR "Labia major" OR "Vagina" OR "vagina" OR "vaginas" OR "vaginal")) OR ("vaginal microbial communities" OR "vaginal microbial community" OR "vaginal microbial composition" OR "vaginal microbial diversity" OR "vaginal microbial ecology" OR "vaginal microbial ecosystem" OR "vaginal microbial flora" OR "vaginal microbiological ecosystem" OR "vaginal microbiological flora" OR "vaginal microbiology" OR "vaginal microbiome" OR "vaginal microbiomes" OR "vaginal microbiota" OR "vaginal microbiotas")) AND ("Lichen Sclerosus et Atrophicus" OR "Lichen sclerosus" OR "Lichen planus" OR "Lichen planus" OR "Penile Tumor" OR "Anus Tumor" OR exp"Vulva Tumor" OR "Vagina Tumor" OR "Uterus Tumor" OR "cancer" OR "cancers" OR "carcinoma" OR "carcinomas" OR "adenocarcinoma" OR "adenocarcinomas" OR "tumor" OR "tumour" OR "tumors" OR "tumours" OR "neoplasm" OR "neoplasms" OR "malignancy" OR "malignancies" OR "Papillomavirus Infection" OR Papillomavir* OR "HPV" OR HPV* OR "HSIL" OR "uvin" OR "dvin" OR "VaIN" OR intraepithel* OR "intra-epithel*" OR "CIN" OR "AIN" OR "Condyloma Acuminatum" OR "Condylomata Acuminata" OR "genital wart" OR "genital warts" OR "Venereal Warts" OR "Venereal Wart" OR "low-

grade SIL" OR "LSIL")) OR (("Microbiome" OR "microbiota" OR microbiota* OR "microbiome" OR microbiom* OR "Microbial Community" OR "Microbial Communities" OR "Microbial Consortia" OR "Mycobiome" OR Mycobiom* OR "Fungal Community" OR "Fungal Communities" OR "bacterial flora" OR "microbial flora") AND ("Vulva" OR "vulva" OR "vulvas" OR "vulvar" OR vulva* OR "Bartholin's Glands" OR "Bartholin's Gland" OR "Clitoris" OR "Labia minora" OR "Labia minor" OR "Labia majora" OR "Labia major"))):ti,ab,kw

Emcare

(((((exp *"Microbiome"/ OR "microbiota".ti,ab OR microbiota*.ti,ab OR "microbiome".ti,ab OR microbiom*.ti,ab OR "Microbial Community".ti,ab OR "Microbial Communities".ti,ab OR "Microbial Consortia".ti,ab OR "Mycobiome".ti,ab OR Mycobiom*.ti,ab OR "Fungal Community".ti,ab OR "Fungal Communities".ti,ab OR *"bacterial flora"/ OR "bacterial flora".ti,ab OR "microbial flora".ti,ab) AND (exp *"Vulva"/ OR "vulva".ti,ab OR "vulvas".ti,ab OR "vulvar".ti,ab OR vulva*.ti,ab OR "Bartholin's Glands".ti,ab OR "Bartholin's Gland".ti,ab OR "Clitoris".ti,ab OR "Labia minora".ti,ab OR "Labia minor".ti,ab OR "Labia majora".ti,ab OR "Labia major".ti,ab OR exp "Vagina"/ OR "vagina".ti,ab OR "vaginas".ti,ab OR "vaginal".ti,ab)) OR "vaginal microbial communities".ti,ab OR "vaginal microbial community".ti,ab OR "vaginal microbial composition".ti,ab OR "vaginal microbial diversity".ti,ab OR "vaginal microbial ecology".ti,ab OR "vaginal microbial ecosystem".ti,ab OR "vaginal microbial flora".ti,ab OR "vaginal microbiological ecosystem".ti,ab OR "vaginal microbiological flora".ti,ab OR "vaginal microbiology".ti,ab OR "vaginal microbiome".ti,ab OR "vaginal microbiomes".ti,ab OR "vaginal microbiota".ti,ab OR "vaginal microbiotas".ti,ab) AND (*"Lichen Sclerosus et Atrophicus"/ OR "Lichen sclerosus".ti,ab OR *"Lichen planus"/ OR "Lichen planus".ti,ab OR exp *"Penile Tumor"/ OR exp *"Anus Tumor"/ OR exp *"Vulva Tumor"/ OR exp *"Vagina Tumor"/ OR exp *"Uterus Tumor"/ OR "cancer".ti,ab OR "cancers".ti,ab OR "carcinoma".ti,ab OR "carcinomas".ti,ab OR "adenocarcinoma".ti,ab OR "adenocarcinomas".ti,ab OR "tumor".ti,ab OR "tumour".ti,ab OR "tumors".ti,ab OR "tumours".ti,ab OR "neoplasm".ti,ab OR "neoplasms".ti,ab OR "malignancy".ti,ab OR "malignancies".ti,ab OR exp *"Papillomavirus Infection"/ OR Papillomavir*.ti,ab OR "HPV".ti,ab OR HPV*.ti,ab OR "HSIL".ti,ab OR "uvin".ti,ab OR "dvin".ti,ab OR "VaIN".ti,ab OR intraepithel*.ti,ab OR intra-epithel*.ti,ab OR "CIN".ti,ab OR "AIN".ti,ab OR exp *"Condyloma Acuminatum"/ OR "Condylomata Acuminata".ti,ab OR "genital wart".ti,ab OR "genital warts".ti,ab OR "Venereal Warts".ti,ab OR "Venereal Wart".ti,ab OR "low-grade SIL".ti,ab OR "LSIL".ti,ab)) OR ((exp *"Microbiome"/ OR "microbiota".ti,ab OR microbiota*.ti,ab OR "microbiome".ti,ab OR microbiom*.ti,ab OR "Microbial Community".ti,ab OR "Microbial Communities".ti,ab OR "Microbial Consortia".ti,ab OR "Mycobiome".ti,ab OR Mycobiom*.ti,ab OR "Fungal Community".ti,ab OR "Fungal Communities".ti,ab OR *"bacterial flora"/ OR "bacterial flora".ti,ab OR "microbial flora".ti,ab) AND (exp *"Vulva"/ OR "vulva".ti,ab OR "vulvas".ti,ab OR "vulvar".ti,ab OR vulva*.ti,ab OR "Bartholin's Glands".ti,ab OR "Bartholin's Gland".ti,ab OR "Clitoris".ti,ab OR "Labia minora".ti,ab OR "Labia minor".ti,ab OR "Labia majora".ti,ab OR "Labia major".ti,ab)))

Academic Search Premier

((((TI("Microbiome" OR "microbiota" OR microbiota* OR "microbiome" OR microbiom* OR "Microbial Community" OR "Microbial Communities" OR "Microbial Consortia" OR "Mycobiome" OR Mycobiom* OR "Fungal Community" OR "Fungal Communities" OR "bacterial flora" OR "microbial flora") AND TI("Vulva" OR "vulva" OR "vulvas" OR "vulvar" OR vulva* OR "Bartholin's Glands" OR "Bartholin's Gland" OR "Clitoris" OR "Labia minora" OR "Labia minor" OR "Labia majora" OR "Labia major" OR "Vagina" OR "vagina" OR "vaginas" OR "vaginal")) OR TI("vaginal microbial communities" OR "vaginal microbial community" OR "vaginal microbial composition" OR "vaginal microbial diversity" OR "vaginal microbial ecology" OR "vaginal microbial ecosystem" OR "vaginal microbial flora" OR "vaginal microbiological ecosystem" OR "vaginal microbiological flora" OR "vaginal microbiology" OR "vaginal microbiome" OR "vaginal microbiomes" OR "vaginal microbiota" OR "vaginal microbiotas")) AND TI("Lichen Sclerosus et Atrophicus" OR "Lichen sclerosus" OR "Lichen planus" OR "Lichen planus" OR "Penile Tumor" OR "Anus Tumor" OR exp"Vulva Tumor" OR "Vagina Tumor" OR "Uterus Tumor" OR "cancer" OR "cancers" OR "carcinoma" OR "carcinomas" OR "adenocarcinoma" OR "adenocarcinomas" OR "tumor" OR "tumour" OR "tumors" OR "tumours" OR "neoplasm" OR "neoplasms" OR "malignancy" OR "malignancies" OR "Papillomavirus Infection" OR Papillomavir* OR "HPV" OR HPV* OR "HSIL" OR "uvin" OR "dvin" OR "VaIN" OR intraepithel* OR "intra-epithel*" OR "CIN" OR "AIN" OR "Condyloma Acuminatum" OR "Condylomata Acuminata" OR "genital wart" OR "genital warts" OR "Venereal Warts" OR "Venereal Wart" OR "low-grade SIL" OR "LSIL")) OR (TI("Microbiome" OR "microbiota" OR microbiota* OR "microbiome" OR microbiom* OR "Microbial Community" OR "Microbial Communities" OR "Microbial Consortia" OR "Mycobiome" OR Mycobiom* OR "Fungal Community" OR "Fungal Communities" OR "bacterial flora" OR "microbial flora") AND TI("Vulva" OR "vulva" OR "vulvas" OR "vulvar" OR vulva* OR "Bartholin's Glands" OR "Bartholin's Gland" OR "Clitoris" OR "Labia minora" OR "Labia minor" OR "Labia majora" OR "Labia major"))) OR ((((KW("Microbiome" OR "microbiota" OR microbiota* OR "microbiome" OR microbiom* OR "Microbial Community" OR "Microbial Communities" OR "Microbial Consortia" OR "Mycobiome" OR Mycobiom* OR "Fungal Community" OR "Fungal Communities" OR "bacterial flora" OR "microbial flora") AND KW("Vulva" OR "vulva" OR "vulvas" OR "vulvar" OR vulva* OR "Bartholin's Glands" OR "Bartholin's Gland" OR "Clitoris" OR "Labia minora" OR "Labia minor" OR "Labia majora" OR "Labia major" OR "Vagina" OR "vagina" OR "vaginas" OR "vaginal")) OR KW("vaginal microbial communities" OR "vaginal microbial community" OR "vaginal microbial composition" OR "vaginal microbial diversity" OR "vaginal microbial ecology" OR "vaginal microbial ecosystem" OR "vaginal microbial flora" OR "vaginal microbiological ecosystem" OR "vaginal microbiological flora" OR "vaginal microbiology" OR "vaginal microbiome" OR "vaginal microbiomes" OR "vaginal microbiota" OR "vaginal microbiotas")) AND KW("Lichen Sclerosus et Atrophicus" OR "Lichen sclerosus" OR "Lichen planus" OR "Lichen planus" OR "Penile Tumor" OR "Anus Tumor" OR exp"Vulva Tumor" OR

"Vagina Tumor" OR "Uterus Tumor" OR "cancer" OR "cancers" OR "carcinoma" OR "carcinomas" OR "adenocarcinoma" OR "adenocarcinomas" OR "tumor" OR "tumour" OR "tumors" OR "tumours" OR "neoplasm" OR "neoplasms" OR "malignancy" OR "malignancies" OR "Papillomavirus Infection" OR Papillomavir* OR "HPV" OR HPV* OR "HSIL" OR "uvin" OR "dvin" OR "VaIN" OR intraepithel* OR "intra-epithel*" OR "CIN" OR "AIN" OR "Condyloma Acuminatum" OR "Condylomata Acuminata" OR "genital wart" OR "genital warts" OR "Venereal Warts" OR "Venereal Wart" OR "low-grade SIL" OR "LSIL")) OR (KW("Microbiome" OR "microbiota" OR microbiota* OR "microbiome" OR microbiom* OR "Microbial Community" OR "Microbial Communities" OR "Microbial Consortia" OR "Mycobiome" OR Mycobiom* OR "Fungal Community" OR "Fungal Communities" OR "bacterial flora" OR "microbial flora") AND KW("Vulva" OR "vulva" OR "vulvas" OR "vulvar" OR vulva* OR "Bartholin's Glands" OR "Bartholin's Gland" OR "Clitoris" OR "Labia minora" OR "Labia minor" OR "Labia majora" OR "Labia major"))) OR ((((SU("Microbiome" OR "microbiota" OR microbiota* OR "microbiome" OR microbiom* OR "Microbial Community" OR "Microbial Communities" OR "Microbial Consortia" OR "Mycobiome" OR Mycobiom* OR "Fungal Community" OR "Fungal Communities" OR "bacterial flora" OR "microbial flora") AND SU("Vulva" OR "vulva" OR "vulvas" OR "vulvar" OR vulva* OR "Bartholin's Glands" OR "Bartholin's Gland" OR "Clitoris" OR "Labia minora" OR "Labia minor" OR "Labia majora" OR "Labia major" OR "Vagina" OR "vagina" OR "vaginas" OR "vaginal")) OR SU("vaginal microbial communities" OR "vaginal microbial community" OR "vaginal microbial composition" OR "vaginal microbial diversity" OR "vaginal microbial ecology" OR "vaginal microbial ecosystem" OR "vaginal microbial flora" OR "vaginal microbiological ecosystem" OR "vaginal microbiological flora" OR "vaginal microbiology" OR "vaginal microbiome" OR "vaginal microbiomes" OR "vaginal microbiota" OR "vaginal microbiotas")) AND SU("Lichen Sclerosus et Atrophicus" OR "Lichen sclerosus" OR "Lichen planus" OR "Lichen planus" OR "Penile Tumor" OR "Anus Tumor" OR exp"Vulva Tumor" OR "Vagina Tumor" OR "Uterus Tumor" OR "cancer" OR "cancers" OR "carcinoma" OR "carcinomas" OR "adenocarcinoma" OR "adenocarcinomas" OR "tumor" OR "tumour" OR "tumors" OR "tumours" OR "neoplasm" OR "neoplasms" OR "malignancy" OR "malignancies" OR "Papillomavirus Infection" OR Papillomavir* OR "HPV" OR HPV* OR "HSIL" OR "uvin" OR "dvin" OR "VaIN" OR intraepithel* OR "intra-epithel*" OR "CIN" OR "AIN" OR "Condyloma Acuminatum" OR "Condylomata Acuminata" OR "genital wart" OR "genital warts" OR "Venereal Warts" OR "Venereal Wart" OR "low-grade SIL" OR "LSIL")) OR (SU("Microbiome" OR "microbiota" OR microbiota* OR "microbiome" OR microbiom* OR "Microbial Community" OR "Microbial Communities" OR "Microbial Consortia" OR "Mycobiome" OR Mycobiom* OR "Fungal Community" OR "Fungal Communities" OR "bacterial flora" OR "microbial flora") AND SU("Vulva" OR "vulva" OR "vulvas" OR "vulvar" OR vulva* OR "Bartholin's Glands" OR "Bartholin's Gland" OR "Clitoris" OR "Labia minora" OR "Labia minor" OR "Labia majora" OR "Labia major"))) OR ((((AB("Microbiome" OR "microbiota" OR microbiota* OR "microbiome" OR microbiom* OR "Microbial Community" OR "Microbial Communities" OR "Microbial Consortia" OR "Mycobiome" OR Mycobiom* OR "Fungal Community" OR "Fungal Communities" OR "bacterial flora" OR "microbial flora") AND AB("Vulva" OR "vulva" OR "vulvas" OR "vulvar" OR vulva* OR

"Bartholin's Glands" OR "Bartholin's Gland" OR "Clitoris" OR "Labia minora" OR "Labia minor" OR "Labia majora" OR "Labia major" OR "Vagina" OR "vagina" OR "vaginas" OR "vaginal")) OR AB("vaginal microbial communities" OR "vaginal microbial community" OR "vaginal microbial composition" OR "vaginal microbial diversity" OR "vaginal microbial ecology" OR "vaginal microbial ecosystem" OR "vaginal microbial flora" OR "vaginal microbiological ecosystem" OR "vaginal microbiological flora" OR "vaginal microbiology" OR "vaginal microbiome" OR "vaginal microbiomes" OR "vaginal microbiota" OR "vaginal microbiotas")) AND AB("Lichen Sclerosus et Atrophicus" OR "Lichen sclerosus" OR "Lichen planus" OR "Lichen planus" OR "Penile Tumor" OR "Anus Tumor" OR exp"Vulva Tumor" OR "Vagina Tumor" OR "Uterus Tumor" OR "cancer" OR "cancers" OR "carcinoma" OR "carcinomas" OR "adenocarcinoma" OR "adenocarcinomas" OR "tumor" OR "tumour" OR "tumors" OR "tumours" OR "neoplasm" OR "neoplasms" OR "malignancy" OR "malignancies" OR "Papillomavirus Infection" OR Papillomavir* OR "HPV" OR HPV* OR "HSIL" OR "uvin" OR "dvin" OR "VaIN" OR intraepithel* OR "intra-epithel*" OR "CIN" OR "AIN" OR "Condyloma Acuminatum" OR "Condylomata Acuminata" OR "genital wart" OR "genital warts" OR "Venereal Warts" OR "Venereal Wart" OR "low-grade SIL" OR "LSIL")) OR (AB("Microbiome" OR "microbiota" OR microbiota* OR "microbiome" OR microbiom* OR "Microbial Community" OR "Microbial Communities" OR "Microbial Consortia" OR "Mycobiome" OR Mycobiom* OR "Fungal Community" OR "Fungal Communities" OR "bacterial flora" OR "microbial flora") AND AB("Vulva" OR "vulva" OR "vulvas" OR "vulvar" OR vulva* OR "Bartholin's Glands" OR "Bartholin's Gland" OR "Clitoris" OR "Labia minora" OR "Labia minor" OR "Labia majora" OR "Labia major")))
