# Supplementary material for: The Human Vulvar Microbiome: A Systematic Review
Source: Microorganisms. 2021 Dec 12;9(12):2568. doi: 10.3390/microorganisms9122568 (PMC8705571; doi:10.3390/microorganisms9122568)
Supplement: Supplementary file 1 [file microorganisms-09-02568-s001.zip › Supplementary Table 1_Summary of lifestyle rules.pdf]

Table S1. Summary of lifestyle rules.

	Author, year, ref	Washing / bathing	Medication / antibiotic use	Menstrual cycle/hormonal status/contraceptive medication	Other relevant in- or exclusion criteria and lifestyle rules
Health and the influence of patient factors	Brown et al, 2007	Standardized soap 1 month prior to sampling.<br><br>No bathing 2 hours prior to sampling.	No antibiotic use 6 weeks prior to study.	Self-reported regular menstrual cycle.<br><br>No report on cycle timing at sampling.	No vulvar, thigh or buttock piercing.<br><br>Standardized catamenial pads used 1 month prior to sampling.<br><br>No sexual intercourse 48 hours prior to sampling.
	Bruning et al, 2020	No showering or bathing during morning of study visit (no exact time frame defined).	No antibiotic use 3 months prior to study.	Post-menopausal subjects were excluded.<br><br>No change of contraceptive medication 3 months prior to study.<br><br>No report on cycle timing at sampling.	Same soap and laundry detergent use during the study.
	Miyamoto et al, 2013	Not described.	No antibiotic use 2 weeks prior to sampling.<br><br>No medicated products on genitalia 2 weeks prior to sampling.	Regular menstrual cycle.<br><br>No report on cycle timing at sampling.	No severe napkin dermatitis in genital area.
	Costello et al, 2009	Bathing on morning of sampling using own hygiene products.	No antibiotic use 6 months prior to sampling.	Not described.	No vulva-specific criteria described.
	Shiraishi et al, 2010	Subjects instructed to use the same soap.	No antibiotic use 6 weeks prior to sampling.	Premenstrual sample obtained in the 1 week prior to expected menstruation.<br><br>Menstrual sample obtained 2 days after menstruation began.<br><br>Subjects instructed to use the same menstrual tools.	No vulvar, thigh or buttock piercing.<br><br>Subjects instructed to use the same underwear.<br><br>Regular sexual intercourse during the examination period.
	Hickey et al, 2015	No bubble baths 48h before sampling.	No antibiotic use 60 days prior to study.	After menarche: sampling visits scheduled not to coincide with menses.	No genital cleansing wipes 48h before sampling.

	Vongsa et al, 2019	No washing, bathing, exercise or swimming 4 hours prior to sampling.	No antibiotics or antifungals 6 weeks prior to sampling.<br><br>No douching, vaginal medication, suppositories, lubricants 48 hours prior to sampling.	Regular menstrual cycle (26-35 days).<br><br>Sampling performed at cycle day 14-21.	No hair-removal in the study area 72 hours prior to sampling.<br><br>No sexual intercourse 48 hours prior to sampling.
Disease	Jayaram et al, 2014	Not described.	Not described.	None of the subjects used oral contraceptives.<br><br>No report on cycle timing at sampling.	None described.
	Murina et al, 2020	Not described.	No antibiotic or antifungal use 14 days prior to sampling.	Not described.	None described.
	Chattopadhyay et al, 2021	Not described.	No topical or systemic antibiotic or steroids use 6 months prior to sampling.	Pre-menarchal girls.	None described.
